# Effects of *Bacillus subtilis* BSNK-5-Fermented Soymilk on the Gut Microbiota by In Vitro Fecal Fermentation

**DOI:** 10.3390/foods11213501

**Published:** 2022-11-03

**Authors:** Yaxin Gao, Lizhen Hou, Miao Hu, Danfeng Li, Zhiliang Tian, Wei Wen, Bei Fan, Shuying Li, Fengzhong Wang

**Affiliations:** 1 Institute of Food Science and Technology, Chinese Academy of Agricultural Sciences, No. 2 Yuan Ming Yuan West Road, Beijing 100193, China; 2 Key Laboratory of Agro-Products Quality and Safety Control in Storage and Transport Process, Ministry of Agriculture and Rural Affairs, Chinese Academy of Agricultural Sciences, Beijing 100193, China; 3 Key Laboratory of Agro-Products Processing, Ministry of Agriculture and Rural Affairs, Chinese Academy of Agricultural Sciences, Beijing 100193, China

**Keywords:** soymilk, *Bacillus subtilis* fermentation, short-chain fatty acids, gut microbiota

## Abstract

The gut microbiota of soymilk intervention is beneficial to maintaining human health. *Bacillus subtilis* fermented soymilk has brought much interest, due to its richness of thrombolytic nattokinase and the strain of potential probiotic properties. In this study, soymilk was fermented by *B. subtilis* BSNK-5, and the BSNK-5-fermented soymilk (SMF) on the production of short chain fatty acids (SCFAs) and the regulation of fecal microbiota was initially evaluated by in vitro fecal fermentation. SMF supplementation obviously increased the levels of SCFAs from 32.23 mM to 49.10 mM, especially acetic acid, propionic acid, and isobutyric acid. Additionally, SMF changed the composition and microbial diversity of gut microbiota. After 24 h of anaerobic incubation in vitro, SMF decreased the *Firmicutes*/*Bacteroidota* ratio favoring weight loss, increased *Lachnospiraceae*_UCG-004 and the other beneficial bacteria producing SCFAs, as well as suppressing pathogenic *Streptococcus* genus. These results revealed the potential use of BSNK-5-fermented soymilk as a potential candidate to promote gut health.

## 1. Introduction

With the rising demand for plant-based foods and the increasing number of vegetarians, soybean has received increasing attention from researchers due to its richness in high-quality protein, lipid, isoflavones, and oligosaccharides. Soybean has been idealized as an important substrate for the development of plant-based functional foods [1,2,3]. Notably, soymilk is an essential soy food in both the America and traditional soy-consuming Asian nations [4,5]. Due to its similar composition to milk, it acts as an excellent plant-derived beverage as a milk substitute in the daily diet, which meets the nutritional needs of people suffering from lactose intolerance and milk allergy [2,6,7]. However, commercial soymilk products exhibit nutritional imbalances, such as the lack of water-soluble vitamins (menadione, riboflavin) and the richness of large molecules (protein), which may not be conducive to a special population with vegetarian needs, or slow metabolism and digestion [7]. Therefore, it is an extremely important to extend the traditional soymilk further to nutraceutical soymilk, to accommodate the current consumption trends.

Microbial fermentation is a reasonable and environmental-friendly way to improve soymilk nutrition and make up for its quality defects [5,8,9]. Several studies have estimated that the soymilk fermented by *Bacillus subtilis* significantly enriches nattokinase with efficient thrombolysis, which served as a dietary therapy to prevent thrombosis [10]. Several *B. subtilis* strains are considered as strains of potential probiotic properties, being aerobic and abundant in proteases, so the soymilk environment is more conducive to its growth, metabolizing large molecules in soymilk and improving the nutrient density of soymilk [11]. Specifically, its fermentation can improve the digestibility of soybean proteins, boost the health benefits of aglycone isoflavones, form various health molecules of vitamins, as well as reduce carbohydrates-induced intestinal flatulence, and positively regulate the gut commensals favoring intestinal absorption [12,13,14].

Gut microbiota created a diverse and complex ecosystem in the intestinal lumen to maintain host intestinal health and homeostasis, including signaling pathways regulation, immune system development, pathogen defense, and autochthonous microbe overgrowth prevention [15]. Conversely, the dysbiosis of the gut microbiota might cause behavioral perturbations, metabolic diseases (e.g., obesity, diabetes), cancers and inflammatory bowel disease [16,17]. In addition, gut microbiota produces specific biological enzymes that convert non-digestible carbohydrates into absorbable short-chain fatty acids (SCFAs) [18,19,20]. SCFAs can lower the pH in the intestinal environment, effectively inhibiting the growth of pathogens, enhancing the epithelial barrier, and modulating immune responses to prevent inflammation and colorectal carcinogenesis [19,21]. It has been shown that diet, in both long-term and short-term exposure, emerged as the major factor in forming the gut microbiota [22,23]. Existing studies have demonstrated that the intake of soymilk obviously conducted to gastrointestinal health, containing the improvement of immune function and intestinal inflammation [24,25,26]. However, little is known about the interaction of *B. subtilis* fermented soymilk with the human gut microecosystem.

Based on our previous study, *B. subtilis* BSNK-5-fermented soymilk was developed and its metabolomics analysis showed that the quality characteristics (nutrition, function and flavor) of soymilk fermented at 48 h were relatively better [27]. Therefore, the purpose of the research was to explore the effect of BSNK-5-fermented soymilk and conventional soymilk on SCFAs production and the microbial composition using in vitro fecal fermentation. This investigation will provide information for better excavating potential health benefits of *B. subtilis* fermented soymilk.

## 2. Materials and Methods

### 2.1. Preparation of B. subtilis BSNK-5-Fermented Soymilk

Fermented soymilk was prepared by following the operational processes of our previous research [11]. *B. subtilis* BSNK-5, screened and preserved at −80 °C by our laboratory, was activated in LB plate by scratching at 37 °C for 12 h. Then a single colony was picked, and reactivated twice in LB liquid medium at 37 °C, 200 rpm for 12 h. Sterilized soymilk was inoculated with 1.0% (*v*/*v*) reactivated strain, and fermented at 37 °C, 200 rpm for 48 h. The uninoculated and unfermented soymilk (0 h) was the control sample, to exclude any non-biological effects. The samples of unfermented soymilk (SMCK) and 48 h fermented soymilk (SMF) centrifugated at 4 °C, 11,000× *g* for 30 min, and the supernatants were collected for the fecal fermentation in vitro.

### 2.2. Collection and Pretreatment of Fecal Samples

Fresh fecal samples were obtained from six normal-weight and healthy human volunteers (three female, three male), aged 20 to 50 years, from Zhejiang Academy of Agriculture Science, China. All volunteers ate traditional Chinese food, free of metabolic and gastrointestinal diseases, and no history of antibiotics, prebiotics or probiotics within three months prior to sample collection. All volunteers were informed about the experiment, filled out a health form and signed a consent form. This study was approved by the Ethical Committee of the Hangzhou Center for Disease Control and Prevention (No. 202047). The fresh fecal sample (0.3 g) was diluted with 3 mL sterilized anaerobic phosphate buffered saline (0.1 M, pH 7.0), to obtain a 10% (*w*/*v*) fecal suspension. The fecal slurry was vortexed to mix thoroughly, and filtered to remove undigested large particles of food residues in the feces.

### 2.3. Fecal Fermentation of BSNK-5-Fermented Soymilk

Fecal slurry was fermented anaerobically in vitro to simulate changes in intestinal microbiota, according to a reported method with slight modifications [15]. The SMCK and SMF samples (72 μL) and fecal suspension (0.5 mL) were added into the sterilized growth medium (tryptone 10 g/L, yeast extract 2.5 g/L, glucose 8 g/L, L-cysteine hydrochloride 1.0 g/L, NaCl 0.9 g/L, KH_2_PO_4_ 0.45 g /L, K_2_HPO_4_ 0.45 g/L, CaCl_2_·2H_2_O 0.06 g/L, MgSO_4_·7H_2_O 0.09 g/L, heme 2 mL/L, vitamin I 200 μL/L). The mixture was incubated anaerobically (90% nitrogen, 5% carbon dioxide, and 5% hydrogen) at 37 °C for 24 h, and centrifuged at 11,000× *g* for 3 min. And the supernatant and bacterial colony precipitation were stored at −30 °C for SCFAs analysis and DNA extraction.

### 2.4. Determination of SCFAs in Fermentation Broth

The SCFAs was analyzed based on a reported method [28]. The fermented supernatant (0.5 mL) was added to 0.1 mL metaphosphoric acid aqueous solution (2.5%, *w*/*v*), containing 75 mM crotonic acid. The mixture was acidified at −30 °C for 24 h. Then, the mixture was centrifuged and filtered with a 0.22 µm membrane filter. Subsequently, the supernatant and SCFA standards (external standards) were implemented on gas chromatography (GC) with a DB-FFAP column (0.32 mm × 30 m × 0.5 μm, Agilent Technologies, Santa Clara, CA, USA). The operational conditions were set as follows: initial temperature of 70 °C for 3 min, ramping to 180 °C at 15 °C/min, then heating to 240 °C at 40 °C/min maintained for 5 min. The injection port and detector temperatures were both 250 °C. Nitrogen was used as the carrier gas with a constant flow rate at 12 mL/min, and the splitting ratio of 1:8, while the rates of air, hydrogen, and nitrogen in detector were 400 mL/min, 40 mL/min, and 30 mL/min, respectively. The SCFAs concentration in fermented supernatant was analyzed based on standard calibration curves.

### 2.5. Fecal Microbial DNA Extraction and Quantitative Real-Time Polymerase Chain Reaction (qRT-PCR)

Fecal microbial DNA from fermentation samples (SMF, SMCK) was extracted using QIAamp DNA Stool Mini Kit according to the instructions (Qiagen, Hilden, Germany). The concentration of extracted DNA was measured with Nano Drop ND-2000 spectrophotometer (Thermo Fisher Scientific, Wilmington, DE, USA). Bacterial 16S rRNA gene was amplified with primers of 341F (5′-CCTAYGGGRBGCASCAG-3′) and 806R (5′-GGACTACNNGGGTATCTAAT-3′). PCR products randomly verified by gel electrophoresis (2% agarose) and purified through the AxyPrep DNA Gel Extraction Kit (Axygen Biosciences, Union City, CA, USA). Next-generation sequencing was performed on the IlluminaMiseq platform. Raw fastq files were demultiplexed, quality-filtered, clustered with 97% similarity cutoff, and employed in defining OTUs (Operational Taxonomic Units) using Mothur. The OTUs were classified using the SILVA database. Then, alpha diversity, and beta diversity were calculated and displayed using R software. Visual Genomics software was used to analyze the sequencing data.

### 2.6. Statistical Analysis

All data were presented as mean ± standard deviation (SD), and all assays included three parallels. Statistical analyses were performed using one way analysis of variance (ANOVA) and Duncan’s test using SPSS 16.0 software (IBM, Chicago, IL, USA). *p* < 0.05 was defined as a statistically significant difference.

## 3. Results

### 3.1. Effects of SMF on SCFAs Production

**Figure 1:**
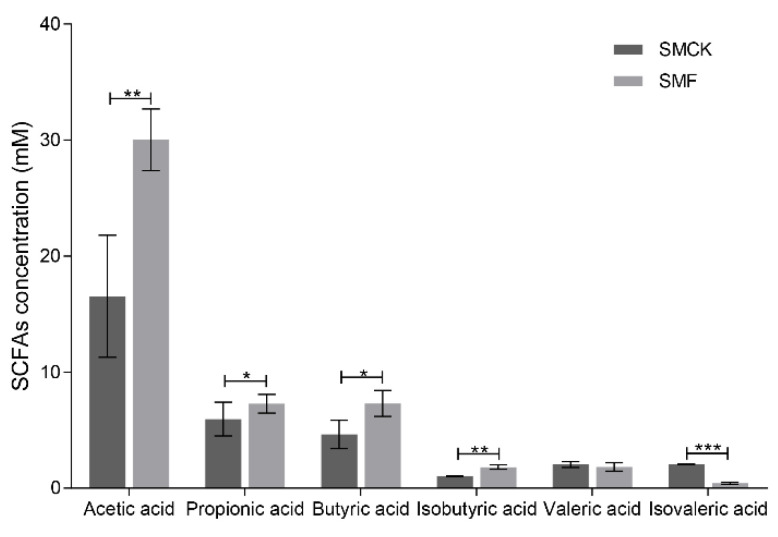
The SCFAs produced by intestinal microbiota after 24 h fermentation. The significantly different results were indicated by specific symbols (*, *p* < 0.05; **, *p* < 0.01; ***, *p* < 0.001).

Generally, the determination of SCFAs is essential to evaluate the properties of BSNK-5-fermented soymilk in vitro fermentation [29]. The concentration of total SCFAs and individual SCFA was shown in Figure 1. The SMF samples (49.10 ± 1.71 mmol/L) constituted higher production of SCFAs than the SMCK samples (32.23 ± 5.82 mmol/L) after the in vitro fermentation. Among them, acetic acid (30.19 ± 2.10 mmol/L) accounted for the largest proportion of SCFAs, followed by propionic acid (7.45 ± 0.69 mmol/L) and butyric acid (7.43 ± 0.93 mmol/L). Compared with the SMCK, acetic acid (*p* < 0.01), propionic acid (*p* < 0.05), butyric acid (*p* < 0.05) and isobutyric acid (*p* < 0.01) showed a significant increase, while isovaleric acid showed a slight decrease (*p* < 0.001) after the treatment of SMF. These results indicated SMF could induce gut bacteria to produce acetic acid, propionic acid and butyric acid.

### 3.2. Effects of SMF on Fecal Microbiota

**Figure 2:**
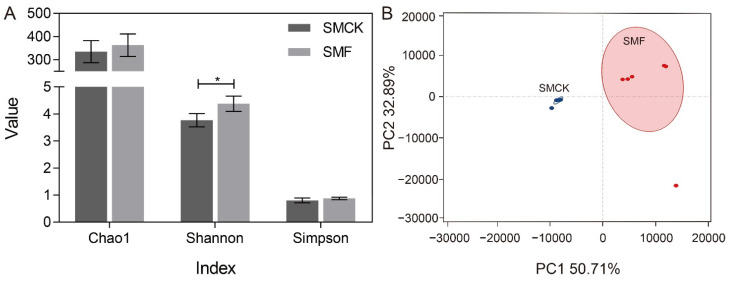
The diversity analysis of microbiota communities after 24 h fermentation. (**A**) Alpha diversity indices (Chao1, Shannon, and Simpson), (**B**) PCA plot. Chao 1 index indicated the community richness, Shannon and Simpson index indicated the community diversity in samples. The significantly different results were indicated by specific symbols (*, *p* < 0.05).

The high-throughput sequencing provided insight into the compositions of the fermented fecal microbiota, which helps elucidate the impact of soymilk fermented by BSNK-5 on microbiota community structures [30,31]. In total, 880,101 clean tags were identified from fecal samples, and each sample had an average of 52,346 valid tags. According to 97% identification threshold, there were 539 operational taxonomical units (OTUs) by clustering the valid tags. Then, alpha diversity was evaluated to measure the intestinal richness and diversity, containing Chao1, Shannon and Simpson index (Figure 2A). The Chao1 index shows the microbial richness in samples. The Shannon and Simpson index show the community diversity [15,32]. After the in vitro fermentation, the Shannon index of SMF samples exhibited higher than that of SMCK group (*p* < 0.05), accompanied by a slight increase in Chao1 and Simpson index. The Shannon index indicated that bacterial diversity was increased. It suggested SMF was conducive to the growth of bacterium and improved the diversity. The principal component analysis (PCA) was utilized to reveal the beta diversity. The first and second axes (PC1: 50.71%, and PC2: 32.89%) interpreted 85.6% of the total variance (Figure 2B). The SMF and SMCK could be clearly separated from each other, indicating SMF caused different clustering of fecal microbiota compositions.

**Figure 3:**
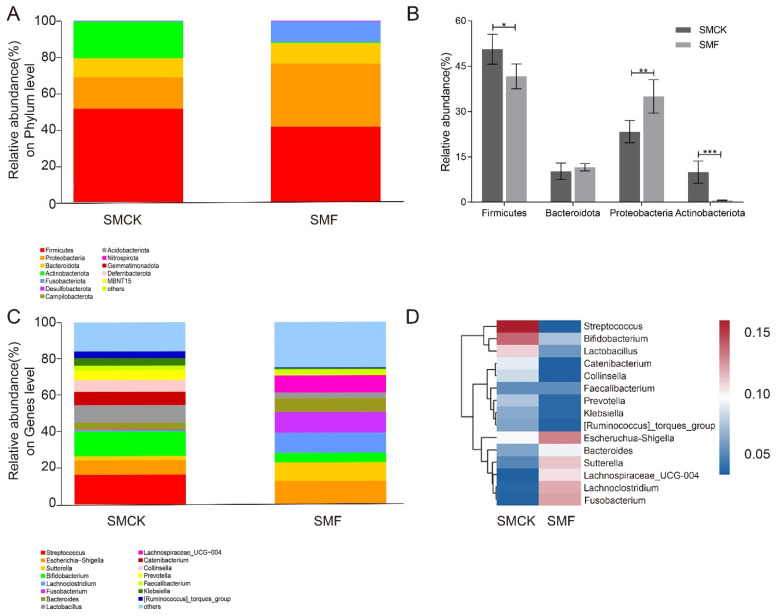
Intestinal microbial community after 24 h fermentation. (**A**) Bar plots of the prevalence at the phylum level, (**B**) the relative abundances of dominant microbiota at the phylum level, the significantly different results were indicated by specific symbols (*, *p* < 0.05; **, *p* < 0.01; ***, *p* < 0.001), (**C**) bar plots of the prevalence at the genus level, (**D**) the heatmap analysis of microbiota community at the genus level. The redder (bluer) the color, the more (less) abundant the species.

The relative abundances of the intestinal microbiota in the SMF and SMCK groups was examined at the levels of phylum and genus. At the phylum level (Figure 3A), the most dominant phylum was *Firmicutes*, followed by *Proteobacteria*, *Bacteroidota*, *Actinobacteriota* and *Fusobacteriota*. When compared to the intestinal microbiota samples in SMCK, *Firmicutes* (41.56%) and *Actinobacteriota* (0.57%) became less abundant. Conversely, *Proteobacteria* (34.68%) and *Fusobacteriota* (11.33%) were significantly more abundant in SMF. The SMF could down-regulate the ratio of *Firmicutes*/*Bacteroidota* from 4.95 to 3.61 (Figure 3B). The relative abundance on the genus level was shown in Figure 3C, the key genus was notably shaped by SMF supplementation, *Streptococcus*, *Collinsella*, *Prevotella*, *Bifidobacterium*, *Lactobacillus* had a notable decrease, while *Lachnospiraceae*_UCG-004, *Bacteroides*, and *Sutterella* were enriched in comparison with SMF and SMCK. Among them, *Streptococcus* was one of the common pathogenic bacteria; *Lachnospiraceae*_UCG-004 was SCFAs-producing bacteria. These results were supported by the heatmap of genus-level abundance (Figure 3D). These results suggested BSNK-5-fermented soymilk was beneficial for the gut microecosystem.

**Figure 4:**
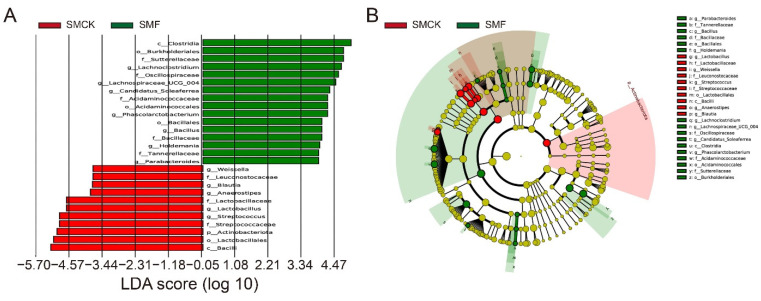
Comparisons of difference microorganisms between the SMCK and SMF groups using LEfSe. (**A**) Linear discriminant analysis (LDA) score, (**B**) LEfSe evolutionary branch graph. The diameter of the node was proportional to the relative abundance. The node in each layer represented the phylum/class/order/family/genus from the inside to the outside, and the annotations of the species labels were opposite; different letters corresponded to different species.

The linear discriminant analysis (LDA) coupled with effect size measurements (LEfSe) was performed to specifically determine the important microbiota between SMF and SMCK groups. As shown in Figure 4A, 27 genera with LDA above 3.0 were identified, exhibited significantly different between SMF and SMCK groups. Based on LDA values, 16 genera were abundant in SMF, and 11 genera in SMCK. Notably, the number of statistically significant OTUs in SMF (color in green) and SMCK (color in red) were 16 and 10, respectively (Figure 4B). In the SMF group, *Clostridia*, *Sutterellaceae*, *Oscillospiraceae* and *Lachnospiraceae* were predominant with higher LDA value, while *Bacilli*, *Lactobacillales* and *Streptococcus* mainly dominated the microbiota of SMCK group, which indicated that the treatment of SMF could notably affect the microbial composition.

### 3.3. Correlation Analysis between SCFAs and Gut Microbiota

**Figure 5:**
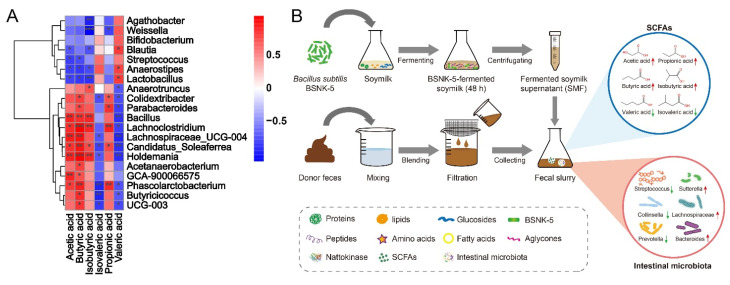
The correlation heatmaps (**A**) and overview (**B**) of gut microbiota and SCFAs. The significantly different results were indicated by specific symbols (*, *p* < 0.05; **, *p* < 0.01; ***, *p* < 0.001).

*B. subtilis* BSNK-5 effectively improved multiple functions of soymilk through fermentation, including the nutritive alteration of protein, and the transformation of isoflavone conformation (glucosides to aglycones) in soymilk, and the enrichment of functional substances [11,27]. Based on the in vitro fermentation of SMF, the production of SCFAs and the structure of gut microbiota had undergone an overall change. To further investigate the influence of gut microbiota community in detail on gas and SCFAs production, the analysis of the differential OTUs between SMCK and SMF was carried out. As shown in Figure 5, the production of SCFAs were mainly attributed to *Bacillus*, *Lachnoclostridium*, *Phascolarctobacterium*, *Lachnospiraceae*_UCG-004 and *Holdemania*. Specifically, *Phascolarctobacterium* was associated with butyric acid and propionic acid. And *Lachnospiraceae*_UCG-004 increased the production of acetic acid and butyric acid. It illustrated SMF exerted positive effects on the growth of SCFAs-producing bacteria. These results explained gut microbiota treated with BSNK-5-fermented soymilk produced more SCFAs, compared with conventional soymilk.

## 4. Discussion

Highly nutrient-dense and plant-based foods, such as fermented soymilk, have been developed, to enhance intestinal health by regulating and restoring the homeostasis of the gut microbiota [7]. This study explored the effect of *B. subtilis* BSNK-5-fermented soymilk on gut microbiota by in vitro fecal fermentation. The results showed the BSNK-5-fermented soymilk increased the content of SCFAs and the modulation of gut microbiota.

SCFAs are considered as valid indicators for assessing intestinal health [15]. Their production is related to the composition of intestinal microbiota, which can metabolize carbohydrates (e.g., dietary fiber, polysaccharides) and proteins in soymilk [33,34]. The previous studies revealed that the production of acetic acid, propionic acid was positively correlated with *Bacteroidota*, *Proteobacteria*, *Lachnospiraceae* [32,35]. It was consistent with the increase of acetic acid, propionic acid (Figure 1) and the alteration of microbiota community in SMF (Figure 3). Acetic acid was utilized as a signaling molecule for gut and hepatocytes in gluconeogenesis and lipogenesis, and it also acted on the brain, heart and peripheral tissues, crossing the blood-brain barrier [36]. Consequently, propionic acid enhanced the sensitivity of tissue insulin, improved proximal colonic barrier function, and affected cholesterol metabolism and neurological functions [37,38]. In addition, the butyric acid inhibits cell apoptosis and prevents colonic cancer [39]. Its increase was extremely associated with the higher abundance of its producing bacteria (e.g., *Bacillus*, *Butyricicoccus*, *Lachnospiraceae*, *Lachnoclostridium*) after SMF treatment (Figure 5) in this study.

The supplementation of fermented soybean products can effectively enhance human intestinal health [7,40]. The top human gut microbiota included *Firmicutes*, *Bacteroidota*, *Proteobacteria* and *Actinobacteriota*. Among them, *Firmicutes* and *Bacteroidota* were the two dominant bacterial phyla of total microbiota during in vitro fecal fermentation (Figure 3A,B), which was similar to those of human gut microbiota *in vivo*. In addition, the ratio of *Firmicutes*/*Bacteroidota* has an increase in inflammatory intestinal environment [8]. The ratio is also considered a biological indicator of obesity, and its reduction is inversely proportional to obesity [32,41]. In consistent with this study, the treatment with SMF resulted in 0.26-fold decrease, compared to the SMCK (Figure 3A,B). Similarly, SMF also modulated the microbiota growth at the genus level. *Sutterella* improved glucose tolerance and the efficacy of antidiabetic drugs [42], and *Lachnospiraceae* contributed to infant neurodevelopment and immunity enhancement [43], both of which increased significantly after SMF treatment (Figure 3C,D). Notably, *Streptococcus* and *Collinsella* could induce the increase of intestinal permeability and the expression of inflammatory factors with detrimental effects [44,45,46], both of which significantly decreased with the addition of SMF in this study (Figure 3C,D). The alteration suggested BSNK-5-fermented soymilk had a complex influence on gut microbiota.

As expected, BSNK-5-fermented soymilk could exert a positive effect on the higher generation of SCFAs and the alteration of bacteria. However, owing to the lack of in vivo studies and in vitro simulated digestion, this study was unable to elucidate the effect of fermented soymilk on the intestinal microbiota in the dietary context. In the future, the gut-improving effects in vivo of *B. subtilis* fermented soymilk should be further investigated by more realistic models and accurate recommended daily doses, to lead consumers to novel nutritional interventions with the help of plant-based diets; meanwhile BSNK-5-fermented soymilk is expected to develop as a commercial functional food.

## 5. Conclusions

In short, this study explored the influence of soymilk fermented by *B. subtilis* BSNK-5 on the intestinal microbiota based on the in vitro simulation of intestinal fermentation. The treatment of SMF accumulated SCFAs, especially acetic acid, propionic acid, butyric acid, and exhibited an increase on the diversity of the microbiota community, specifically reducing the ratio of *Firmicutes*/*Bacteroidota*, and stimulating the growth of beneficial and SCFAs-producing bacteria, such as *Lachnospiraceae*_UCG-004. These results illustrated that SMF had complicated effect on gut microbiota. Therefore, this study provided compelling evidence that BSNK-5-fermented soymilk showed potential for the development as a functional food.

## Data Availability

All related data and methods are presented in this paper. Additional inquiries should be addressed to the corresponding author.
